# Correlation of CT imaging and histology to guide bone graft selection in scaphoid non-union surgery

**DOI:** 10.1007/s00402-018-2983-0

**Published:** 2018-07-13

**Authors:** Gernot Schmidle, Hannes Leonhard Ebner, Andrea Sabine Klauser, Josef Fritz, Rohit Arora, Markus Gabl

**Affiliations:** 1 Department of Trauma Surgery, Medical University Innsbruck, Anichstraße 35, 6020 Innsbruck, Austria; 2 Department of Radiology, Medical University Innsbruck, Anichstraße 35, 6020 Innsbruck, Austria; 3 Department of Medical Statistics, Informatics and Health Economics, Medical University Innsbruck, Schöpfstraße 41, 6020 Innsbruck, Austria

**Keywords:** Scaphoid non-union, Bone viability, CT, Histology, Vascularized, Non-vascularized, Bone graft

## Abstract

**Introduction:**

For the treatment of scaphoid non-unions (SNU), different surgical techniques, including vascularized and non-vascularized bone grafts, are applied. Besides stability, vascularity, and the biological situation at the non-union site are important for healing and the appropriate choice of treatment. We assessed the healing potential of SNUs by histological parameters and compared it to CT parameters of bone structure and fracture location. Based on the results, we developed a CT classification and a treatment algorithm to impact graft selection in SNU surgery.

**Patients and methods:**

Preoperative 2D-CT reformations of 29 patients were analyzed for trabecular structure, sclerosis, and fragmentation of the proximal fragment. The fracture location was assessed on 3D-CT reconstructions and grouped in three zones depending on the potential blood supply. Samples were taken during surgery for histological evaluation. Histological parameters of bone healing were defined and a bone healing capacity score (BHC), reflecting histological bone viability, was calculated. CT findings were compared to BHC, age of SNU, and time to union.

**Results:**

Cases with trabecular structure and without fragmentation showed a statistically significant higher BHC. Time to union was significantly faster if trabecular structure was present and sclerosis was absent. In intraarticular proximal pole non-unions, where no blood supply is assumed, the BHC was statistically significantly lower and time to union was longer compared to SNUs of the other locations. A statistically significant correlation between BHC and time to union was found in the proximal and distal fragment with higher BHC associated with faster healing.

**Conclusions:**

CT parameters of bone structure and fracture location can reflect histological healing capacity of SNUs. This can guide bone graft selection in SNU surgery.

## Introduction

The scaphoid is the most frequently fractured carpal bone. Its complex shape and tenuous blood supply increase the risk of scaphoid non-unions (SNU) and, as a consequence, advancing osteoarthritis of the wrist [1–3]. Persistent SNU results in posttraumatic osteoarthritis in 75–97% of cases after 5 years and in 100% after 10 years [4, 5]. The retrograde arterial blood supply from distal makes it likely that the proximal pole can develop avascular necrosis [6].

Different methods can achieve anatomic reconstruction and bone fusion in SNU. These methods are based on the principle of restoring anatomy by correcting deformity and providing stability, viability, and vascularity [7]. The techniques can be grouped into stabilizing procedures using implants and revascularizing procedures using vascularized bone grafts. The use of non-vascularized autologous bone grafts has proven to be effective in specific situations [8–10]. In the case of an avascular proximal fracture fragment, vascularized bone grafting is the recommended treatment [11–14], but there are different opinions on the necessity of vascularized vs. non-vascularized bone grafting [15, 16].

Union rates of up to 90% have been reported, depending on the applied surgical therapy and techniques. They decline significantly in longstanding non-unions. This shows the dynamic nature of SNU reducing their healing potential over time [17].

The ideal choice of surgical therapy still remains a subject of debate [18]. It is based on X-ray, CT, and MRI and accomplished by the intraoperative findings [19–21]. Standard X-rays lack accuracy in reflecting fracture location, fracture patterns of displacement, or stability in general [22, 23]. 2D-CT reformations allow for a better assessment of displacement [24, 25]. 3D-CT reconstructions provide information on the exact location of the fracture line in relation to relevant anatomical landmarks [26].

Apart from anatomical location and stability issues, scaphoid non-union is highly related to the precarious blood supply of the proximal fragment. MRI has shown an ability to predict vascularity of the proximal pole fragment, but the value in predicting bone healing is limited [18, 27, 28]. For scaphoid vascularity, histopathological analysis can be considered the current reference standard [15].

In a previous study, the histological parameters of bone healing were defined and a histological bone healing score (BHC) was calculated and correlated with time intervals after fracture [29].

The primary aim of the present manuscript was to compare the BHC with 2D-CT parameters of bone structure of the proximal fragment (trabecular structure, sclerosis, and fragmentation) and 3D-CT fracture location and to correlate BHC as well as CT parameters with time to union.

Based on the results, the secondary aim was to develop a classification system and a treatment algorithm to guide the use of vascularized and non-vascularized bone grafts in SNU surgery.

## Patients and methods

### Patients

Ethics approval from the relevant research ethics committee and informed consent from all patients included in the study was obtained.

The study sample comprised 29 patients (24 male, 5 female), with a mean age of 28.7 years (15.3–46.4 years) who underwent SNU surgery from June 2011 to June 2016 after failed conservative treatment or occult fractures. Patients with previous surgical intervention or without preoperative CT examination were excluded.

In 26 patients, reconstructive surgery was performed, whereas 3 patients underwent 4 corner fusions as a salvage procedure. 10 patients received a non-vascularized bone graft (NVBG) and in 16 patients a vascularized bone graft (VBG) was used in combination with rigid internal fixation.

### Histology

The histological methodology was presented in detail in the Journal of Anatomy [29].

Samples were taken during operative intervention. Haematoxylin and Eosin (HE), Azan, Toluidine, von Kossa, and Tartrate-resistant acid phosphatase (TRAP) staining were used to characterize the samples histologically. We determined distribution of Collagen 1 and 2 by immunocytochemistry, while scanning electron microscopy (SEM) was employed to investigate the ultrastructure.

The samples from each patient were subdivided into a proximal, gap, and distal section. Histological parameters were selected according to the existing literature [30–32] and to the longstanding experience of our histological experts. Different parameters were defined and evaluated for the presence or absence of each parameter. They were grouped according to their effect on bone healing into parameters with high, partial, and little activity.

Histopathological signs for high bone healing activity included osteoid formation, cell density and cell types, trabecular thickening, the presence of trabecular spikes bordered by osteoblasts (cell lines), neovascularisation, and the presence of collagen 1.

Histopathological signs for bone degeneration consisted of the presence of a sclerotic seal on the fracture site, a dense sclerotic area, few cells, the presence of cysts, the presence of osteoclasts or macrophages, and the presence of a “foam deposit” observed in areas with advanced bone degradation.

Histopathological signs of structure and the consistency of tissue in the gap comprised the presence of blood vessels, status of gap filling, type, and concomitance of collagens.

To quantify the overall biological activity, we calculated the bone healing capacity score (BHC) summarizing the individual parameters. For parameters in the high activity group, every point (presence of parameter) was scored with 2 (as this is evaluated as being better than partial activity). Partial activity was given a score of 1 (as in this case still some healing is taking place), and little activity was awarded a score of − 1 (as this has to be considered negative) [29].

### Preoperative imaging

CT images were acquired with a 64-detector row MDCT scanner (LightSpeed™ VTC, GE Milwaukee) using a standard bone protocol with a slice thickness of 0.625 mm and acquisition parameters of 100 kV and 100 mAs. All CT examinations were performed without a plaster cast.

In general, 2D parameters of bone structure can be evaluated on conventional CT slice images. For our study, we defined the region of interest (ROI) for 2D assessment more precisely to facilitate the measurement of interobserver reliability. In the coronal and sagittal planes, the ROI was centered over the fracture gap and aligned parallel to the fracture line to display the most central part of the proximal fragment using multi-planar reconstructions. For the analysis of SNU location, a 3D volume rendering model of the scaphoid was created.

#### 2D-CT parameters

**Fig. 1:**
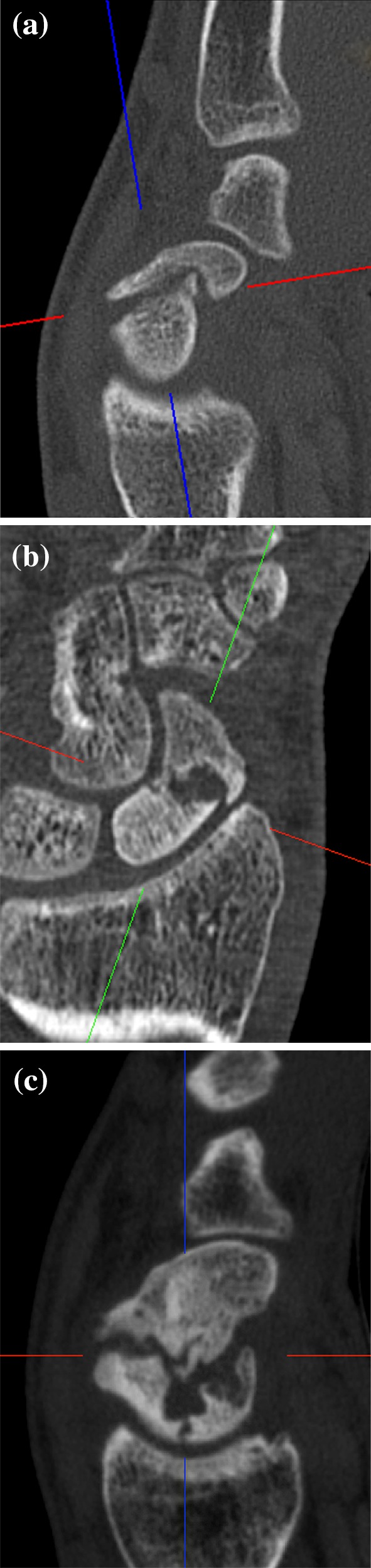
2D-CT parameters of the proximal fragment. **a** Trabecular bone structure. **b** Sclerosis of the proximal fragment in comparison to the distal fragment. **c** Fragmentation of the proximal pole

On 2D-CT reformations, three blinded observers (two senior hand surgeons and one senior radiologist) assessed the following parameters of the proximal fragment:

Trabecular structure was assessed as present if > 50% of the cancellous bone showed signs of typical grid structure compared to the distal fragment (Fig. 1a).

Sclerosis was assessed in comparison to the distal fragment and rated as positive if > 50% of the cancellous part was involved (Fig. 1b).

Fragmentation as a sign of biological and structural breakdown may manifest as partial inner or complete outer fragmentation (Fig. 1c). If fragmentation of cancellous central parts of the proximal fragment exceeded half of the remaining bone and just the outer cortico cartilaginous shelf remained intact, it was defined as inner fragmentation or bone infarction. Outer fragmentation was present if central parts of the articular surface for the radius were separated and the fragmented part measured at least 1/3 of the total size of the proximal fragment. Peripheral wedge fractures and bony ligament avulsions were not rated as fragmentation as these are primary fracture patterns with potential biomechanical or biological impact, but are not signs of necrotic tissue or bone dissolution/infarction.

**Fig. 2:**
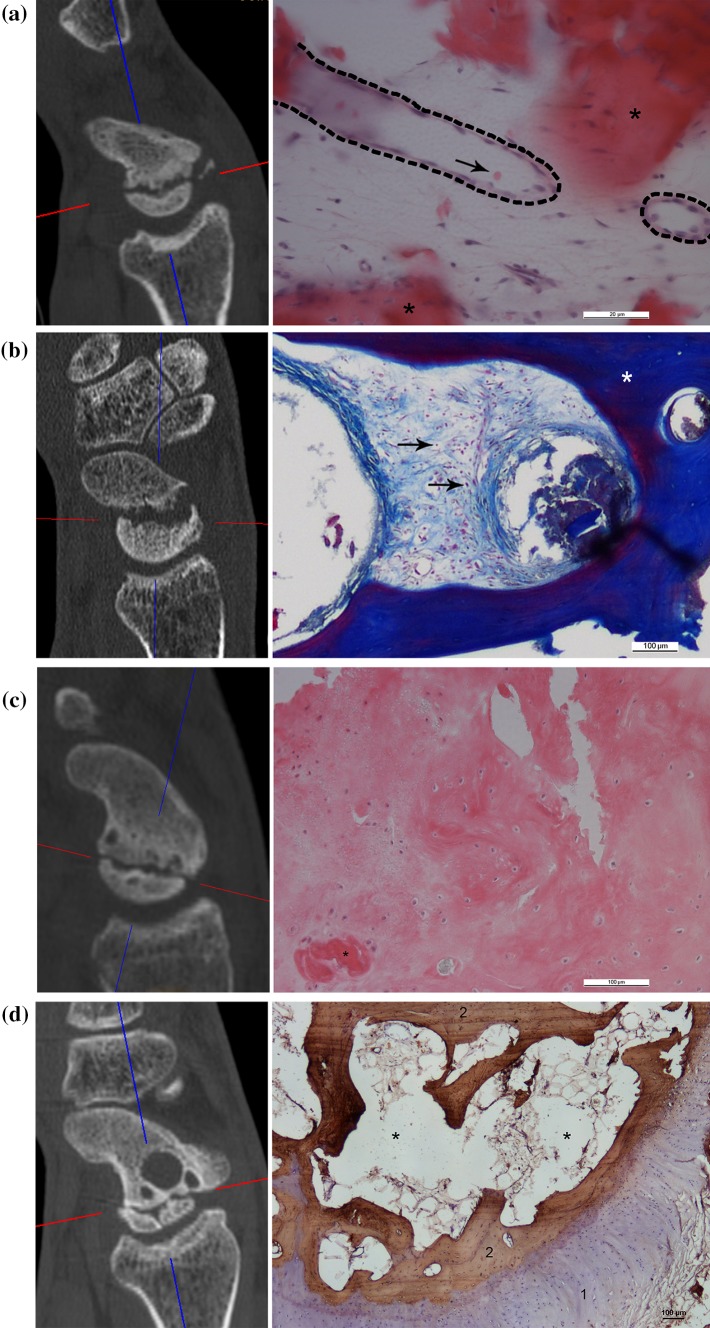
SNU types according to the presence of 2D-CT parameters at the proximal fragment with the corresponding histological image. **a** Type 1: presence of trabecular structure, and absence of sclerosis and fragmentation. The histological image shows blood vessels (dashed lines), singular erythrocytes (red, arrow), and osteoid (red, asterisk) representing highly active tissue; HE stain. Scale bars = 20 µm. **b** Type 2: presence of trabecular structure and sclerosis, and absence of fragmentation. Connective tissue with densely packed cell nuclei (red, arrows) and collagen fibers (blue) next to thickened trabeculae (asterisk) as signs of active tissue; AZAN stain. Scale bars = 200 µm. **c** Type 3: absence of trabecular structure, presence of sclerosis, and absence of fragmentation. A dense sclerotic area, few cell nuclei (purple), and osteoclasts (red, asterisk) represent tissue with less biological activity; HE stain. Scale bars = 100 µm. **d** Type 4: absence of trabecular structure, and presence of sclerosis and fragmentation. The corresponding histological image shows the cartilage shelf (purple, 1) and sparsely trabeculae positive for collagen 1 (brown, 2) with huge empty spaces in between (asterisks) reflecting reduced viability and disintegration of bone structure; TRAP stain and collagen 1. Scale bar = 100 µm

Based on these parameters of bone structure, we defined four subtypes of SNU (Table 1). This classification system ranges from Type 1 representing normal bone structure to Type 4 reflecting bone infarction with complete disintegration of the proximal fragment. The 2D-CT parameters of trabecular structure, sclerosis, and fragmentation, and the corresponding histologic images are shown in Fig. 2.

**Table 1:** Types of SNU according to the presence of 2D-CT parameters of bone structure

2D-CT SNU subtypes	Trabecular structure	Sclerosis	Fragmentation
Type 1 (*n* = 11)	+	−	−
Type 2 (*n* = 9)	+	+	−
Type 3 (*n* = 5)	−	+/−	−
Type 4 (*n* = 4)	−	+	+

*SNU* scaphoid non-union, + presence of respective parameter, − absence of respective parameter

#### 3D-CT fracture location

**Fig. 3:**
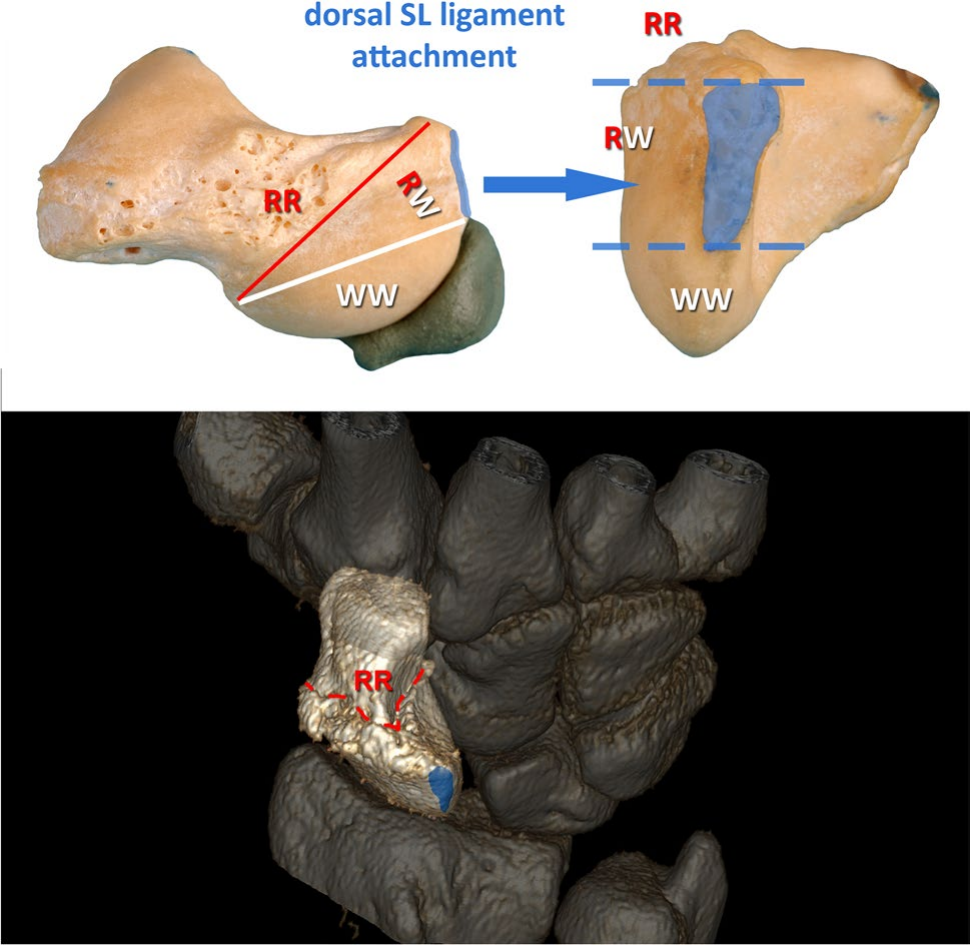
Anatomical specimen of a scaphoid with 3D fracture zones depicted. The dorsal tubercle is the proximal end of the RR zone. SNUs with fracture lines that run through the area of the dorsal SL ligament attachment (blue) are allocated to the RW zone. The WW zone lies proximal to the SL ligament attachment. The 3D reconstruction at the bottom shows a dorsal view of a clinical case with a fracture line (dotted red line) distal to the SL ligament attachment (blue) equaling an injury at the RR zone. *SL* scapholunate, *RR* red/red zone, *RW* red/white zone, *WW* white/white zone

The location of the SNU and the course of the fracture line were related to anatomy and potential blood supply using 3D CT reconstructions. The dorsal interosseous scapholunate ligament attachment was visualized on the 3D model, and depending on the fracture course relative to this anatomical structure the non-unions were divided in three zones (Fig. 3).

SNUs with complete intraarticular cartilaginous sites were defined as white/white zone (WW, proximal), representing anatomical proximal pole non-unions where no separate blood supply can be expected.

Partially, intraarticular fracture lines with some cartilage free areas for ligament attachment and blood supply were allocated to SNUs of the intermediate red/white zone (RW, transition).

In extraarticular fracture lines at the anatomical waist region, a good blood supply of both fragments can be expected. These were defined as SNUs of the red/red zone (RR, waist).

18 cases (62.1%) were found at the completely intraarticular portion of the proximal pole (WW zone) and 11 cases (37.9%) in the other zones (3 RW, 8 RR).

### Interrater reliability

Interrater reliability among the three raters for the 2D-CT parameters of trabecular structure, sclerosis, and fragmentation was assessed using Fleiss’ Kappa (*κ*) [33]. Values less than 0.5 were rated as poor, between 0.5 and 0.75 as moderate, between 0.75 and 0.9 as good, and greater than 0.90 as excellent reliability [34].

### Statistics

Descriptive statistics are expressed as mean values and standard deviations or absolute and relative frequencies.

The Kolmogorov–Smirnov test was used for the determination of normal distribution. We assessed differences in BHC, age of SNU, and time to union related to the presence of 2D-CT parameters and to 3D-CT fracture zones with Student’s *t* tests for independent samples. Pearson correlation coefficients and linear regression analyses were calculated to investigate the relationship between the age of the SNU and the BHC with time to union. We used a significance level of *α* = 0.05 (two sided). Statistical analyses were performed using SPSS, version 24.0 (IBM Corp, Armonk, NY) and R version 3.3.0.

## Results

### Preoperative imaging

#### BHC and 2D CT parameters

Cases with trabecular structure in CT showed higher BHC, reaching statistical significance for the proximal fragment. BHC was significantly higher in cases without fragmentation. The results for the CT parameter of sclerosis did not show statistical significance (Table 2).

**Table 2:** BHC of the proximal fragment, gap, and distal fragment vs. 2D-CT parameters of bone structure at the proximal fragment

	BHC proximal	BHC gap	BHC distal
	Mean ± SD	Mean ± SD	Mean ± SD
*Trabecular structure*			
Yes (*n* = 20)	5.3* ± 3.6	6.3 ± 4.1	6.5 ± 3.6
No (*n* = 9)	0.9* ± 1.9	4.9 ± 4.5	4.3 ± 3.9
* p* values	< 0.001	0.466	0.187
*Sclerosis*			
Yes (*n* = 18)	3.0 ± 3.8	6.4 ± 4.2	5.3 ± 3.4
No (*n* = 11)	5.3 ± 3.5	4.8 ± 4.3	6.6 ± 4.5
* p* values	0.116	0.367	0.439
*Fragmentation*			
Yes (*n* = 4)	1.3* ± 1.9	2.0 ± 4.1	3.0 ± 4.8
No (*n* = 25)	4.3* ± 3.8	6.5 ± 3.9	6.3 ± 3.5
* p* values	0.036	0.111	0.274

*BHC* bone healing capacity score, *SD* standard deviation

*Significant difference between presence and absence of 2D-CT parameters

The age of the SNU had no statistically significant influence on the presence of 2D-CT parameters in general.

The presence of trabecular structure in the proximal fragment led to a statistically significant faster healing, whereas cases with sclerosis showed a statistically significant longer time to union. For the parameter of fragmentation we found no significant differences (Table 3).

**Table 3:** Age of SNU and time to union vs. 2D-CT parameters of bone structure at the proximal fragment

	Age of SNU (months)		Time to union (months)
	Mean ± SD		Mean ± SD
*Trabecular structure*			
Yes (*n* = 20)	48.4 ± 64.7	Yes (*n* = 18)	3.4* ± 0.8
No (*n* = 9)	48.7 ± 56.6	No (*n* = 7)	5.2* ± 0.8
* p* values	0.991		< 0.001
*Sclerosis*			
Yes (*n* = 18)	44.9 ± 49.0	Yes (*n* = 15)	4.3* ± 1.2
No (*n* = 11)	54.4 ± 79.9	No (*n* = 10)	3.4* ± 0.9
* p* values	0.728		0.038
*Fragmentation*			
Yes (*n* = 4)	87.0 ± 69.5	Yes (*n* = 3)	5.0 ± 1.0
No (*n* = 25)	42.3 ± 59.1	No (*n* = 22)	3.8 ± 1.1
* p* values	0.295		0.159

*SNU* scaphoid non-union, *SD* standard deviation

*Significant difference between presence and absence of 2D-CT parameters

#### BHC and 3D CT fracture location

SNUs of the WW zone had significantly lower BHC at the proximal fragment than those of the RW/RR zone. The proximal fragment had significantly worse BHC compared to the other sample locations (Table 4).

**Table 4:** BHC in relation to the 3D fracture zone

3D fracture zone	BHC proximal	BHC gap	BHC distal
	Mean ± SD	Mean ± SD	Mean ± SD
WW (*n* = 18)	2.8* ± 3.7	5.8 ± 4.2	5.2 ± 3.9
RW/RR (*n* = 11)	5.8* ± 3.2	5.7 ± 4.5	7.0 ± 3.6
*p* values	0.036	0.956	0.240

*BHC* bone healing capacity score, *WW* white/white, *RW*/*RR* red/white and red/red, *SD* standard deviation

*Significant difference between 3D fracture zones

Concerning the age of the SNU no statistically significant difference was found.

SNUs at the proximal WW zone showed a statistically significant longer time to union compared to SNUs of the other locations (Table 5).

**Table 5:** Age of SNU and time to union in relation to the 3D fracture zone

3D fracture zone	Age of SNU (months)		Time to union (months)
	Mean ± SD		Mean ± SD
WW (*n* = 18)	56.8 ± 66.8	(*n* = 14)	4.4* ± 1.1
RW/RR (*n* = 11)	34.8 ± 51.0	(*n* = 11)	3.4* ± 0.8
*p* values	0.326		0.016

*SNU* scaphoid non-union, *WW* white/white, *RW*/*RR* red/white and red/red, *SD* standard deviation

*Significant difference between 3D fracture zones

### Bone healing and time to union

Bone healing could be achieved in all but one case.

We observed one case of persistent non-union in a 31.5-year-old male patient with a delayed union (5 months) treated with plating and a NVBG from the iliac crest. The fracture was located in the WW zone and showed the bone structure of a Type 3 SNU with a BHC score of the proximal fragment of 0.

**Fig. 4:**
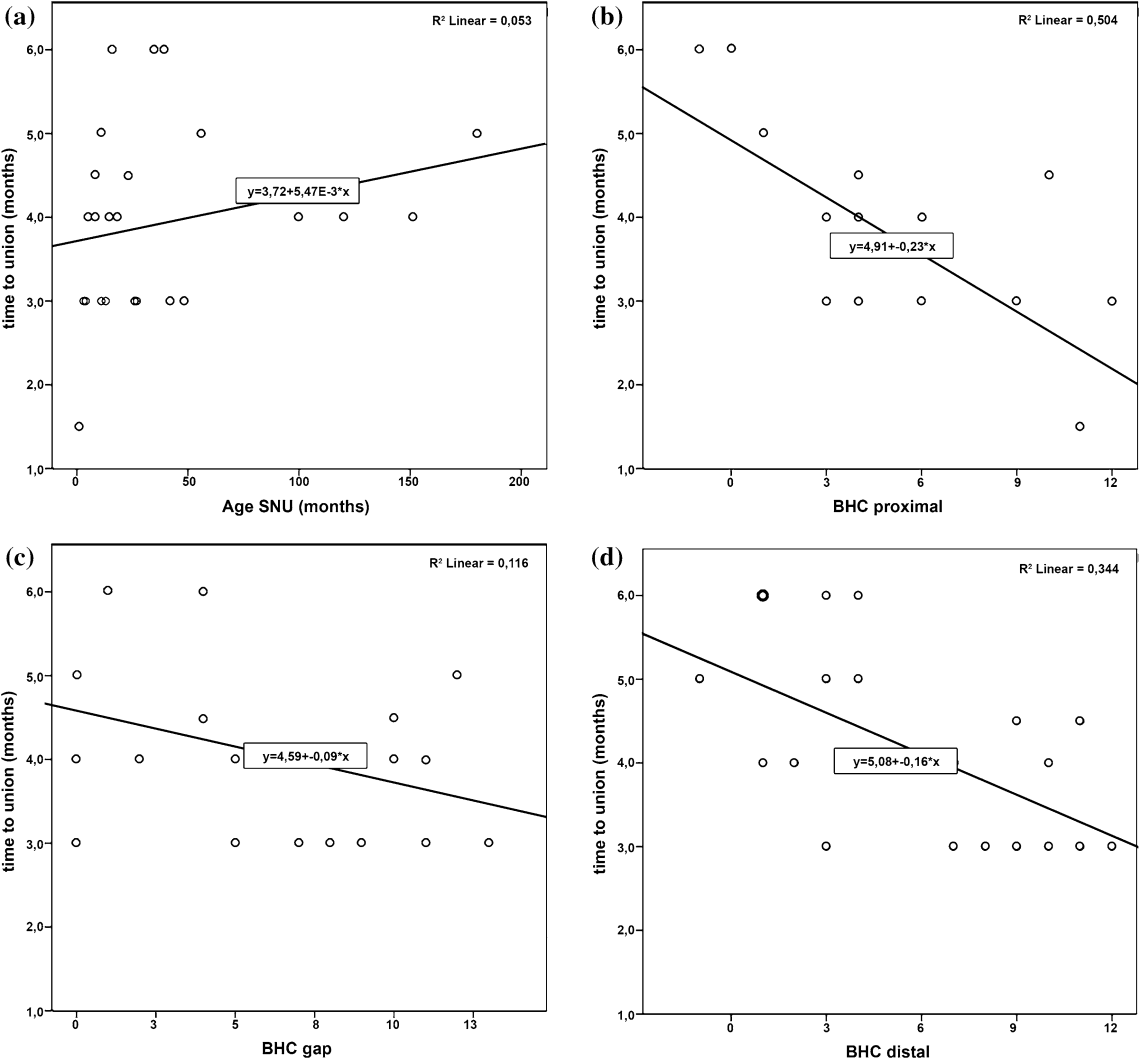
**a** Scatter plot of age of non-union vs. time to union with regression line (*y* = 3.72 + 5.47*E* − 3 × *x*) and *R*^2^ (*R*^2^ = 0.053). **b** Scatter plot of BHC score of the proximal fragment vs. time to union with regression line (*y* = 4.91 − 0.23 × *x*) and *R*^2^ (*R*^2^ = 0.504). **c** Scatter plot of BHC score of the gap section vs. time to union with regression line (*y* = 4.59 − 0.09 × *x*) and *R*^2^ (*R*^2^ = 0.116) without significant correlation. **d** Scatter plot of BHC score of the distal fragment vs. time to union with regression line (*y* = 5.08 − 0.16 × *x*) and *R*^2^ (*R*^2^ = 0.344)

The age of the SNU was statistically not associated with time to union (*r* = 0.231, *p* = 0.266). The analysis of the time to union vs. BHC of the proximal and distal fragment revealed a statistically significant correlation of *r* = 0.710 (*p* < 0.001) and *r* = 0.586 (*p* = 0.003), respectively, with higher BHC being associated with faster healing. In contrast, at the gap region no statistically significant correlation with time to union was found (*r* = 0.341, *p* = 0.120) (Fig. 4).

### Interrater reliability

Good interrater reliability was achieved for the parameters trabecular structure (*κ* = 0.802), sclerosis (*κ* = 0.898), and fragmentation (*κ* = 0.770).

## Discussion

The importance of restoration of stability is widely agreed on and new studies emphasize even the use of two screws to increase rotational stability [35]. The selection of an appropriate bone graft on the other hand remains a disputed topic [36, 37]. Current classifications are helpful tools to assess stability [25, 26], but they do not reflect the biological situation of bone healing [30]. The implementation of histological characterization of SNUs is required to optimize diagnosis and therapy [38]. SNU treatment and the use of vascularized vs. non-vascularized bone grafting is based on vascularity assessment and assumed healing capacity of the scaphoid. However, vascularity alone has not proven to be a clear predictor of SNU healing, and the value of MRI in predicting bone healing is limited [15].

### Vascularized versus non-vascularized bone grafts

There is a wide range of opinions on whether to use vascularized (VBG) or non-vascularized bone grafting (NVBG) for SNU treatment [12, 13, 15]. Whereas structural bone grafts (cortico-cancellous) have proven to be superior in restoring carpal geometry over cancellous-only bone grafts [39], comparisons between NVBG and VBG often lack details on fracture characteristics (location, bone structure, displacement), making it difficult to compare similar groups and, hence, to arrive at clear conclusions. Both vascularized and non-vascularized bone grafts can lead to good healing rates if preoperative workup, risk factor assessment, and patient selection are performed diligently.

NVBGs provide viable tissue that serves as a scaffold. They need biologically active tissue in the remaining bone fragments so they can be replaced and integrated over time by creeping substitution [40]. In longstanding SNUs, higher rates of treatment failure of up to 54% are reported with NVBGs [41]. Whereas the BHC at the proximal fragment showed no correlation to age of the SNU, the increasing compromise of the distal fragment over time might indicate its importance as an engine for creeping substitution [29]. The age of SNU may be used as an additional decision aid [42].

VBGs represent biologically active tissue that support healing efforts directly [14]. VBGs are generally used more often in cases that are more likely to fail and are recommended for avascular necrosis, proximal pole non-union, longstanding SNU, or failed previous surgery [12, 42, 43].

### 2D-CT imaging

CT is superior to standard radiographs and MRI in assessing the morphology (localization, displacement, comminution, instability) in scaphoid fractures and may, therefore, be best suited to provide information about bone structure [44]. Micro CT has been used to depict the changes in bone ultrastructure in detail, but is not available in clinical routine [45].

Trabeculae are a sign of functioning bone homeostasis with a balance of bone formation and resorption. Bone from a non-union compared with normal scaphoid bone is characterized by an increased number of trabeculae on both sides and an increased trabecular thickness and number on the proximal side [45]. The absence of converging trabeculae between the fracture fragments is correlated with histological evidence of avascular necrosis [20]. In our study, the presence of trabecular structure in the proximal fragment on 2D-CT reformations was related to the BHC as a measure of bone viability. This observation was further supported by the faster time to union of cases with trabecular structure as opposed to those without.

The presence of sclerosis has been considered a sign of avascular necrosis [20], even though some studies have shown a lack of correlation [21, 46, 47]. Sclerotic fragments show higher bone volume and trabecular thickness, reducing the surface area. This may reflect decreased metabolic activity of the bone [45]. Our results showed no significant difference in BHC of cases with or without sclerosis, but we found a longer time to union if sclerosis was present.

Sclerotic fragments are not necrotic or dead bone, as necrotic tissue would be expected to disintegrate and be resorbed—as is the case in fragmented proximal poles. Sclerosis may reflect fragment vascularity, but not biological activity levels and potential healing. Nevertheless, it may guide decision making in the selection of an appropriate treatment method. The presence of sclerosis in the proximal fragment may even be beneficial for screw anchorage in the case of SNU surgery.

Fragmentation is seen as the last step of bone degradation if no bone homeostasis can be achieved within the proximal fragment. In our opinion, these are the only cases of a true avascular necrosis that combine lack of vascularity, osseous disintegration, and low BHC. Vascularized osteochondral femoral grafts are used to reconstruct SNUs with fragmented proximal poles [12]. The low BHC score found in our study supports this approach.

### 3D-CT fracture location

Vascular supply is, apart from stability, an important factor for achieving bone union. The location of the fracture plays an important role for the development of an avascular necrosis [4, 30]. We, therefore, defined zones according to anatomical landmarks that are relevant for blood supply as well as stability.

The exact fracture location according to anatomical landmarks is seldom reported. The first introduction of 3D imaging to classify SNU according to anatomical landmarks was done by Nakamura and Moritomo, who distinguished stable and unstable forms of SNU [48, 49].

Our results showed that SNU of the complete intraarticular proximal pole region (WW) had less histologically assessed viability than more distal fractures of the transition zone (RW) or the waist region (RR). The location of the fracture line impacts radiological signs of bone structure and biological healing capacity of SNU with less activity in the WW zone.

### Clinical relevance

**Fig. 5:**
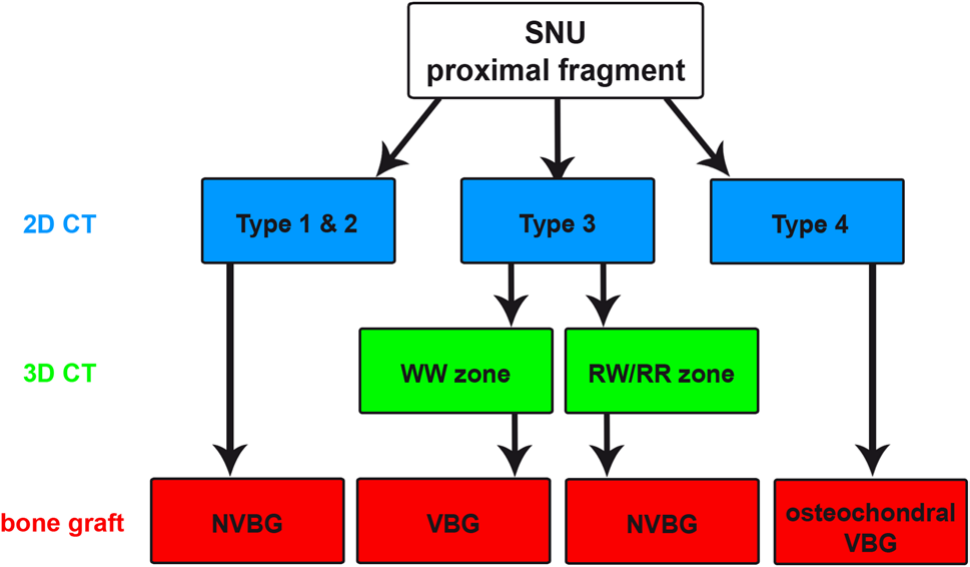
Treatment algorithm for bone graft selection. 2D-CT SNU types (blue), 3D-CT fracture location (green), and recommended bone graft (red)

Based on our results, we developed a treatment algorithm for bone graft selection in SNU surgery combining the assessment of bone structure on 2D-CT images with information on the 3D-CT fracture location (Fig. 5). The first step of the decision tree comprises the assessment of CT parameters of bone structure leading to a clear treatment suggestion for 3 of the 4 SNU types. A 3D-CT assessment of fracture location changes the treatment recommendation only in Type 3 SNU (in our study five cases, 17.2%) and can be limited to these cases.

As long as there is trabecular structure at the proximal fragment (Types 1 and 2), some sort of biological activity is present supporting the use of NVBGs for all SNU locations. The absence of trabecular structure and fragmentation (Type 3) should direct treatment to the use of VBGs at the WW zone, whereas NVBGs can still be considered for SNUs of the RW or RR zone. The presence of fragmentation (Type 4) indicates the worst BHC at the proximal fragment (no anchor point for bridging bone) and should be treated by vascularized osteochondral grafts. The age of SNU may be used as an additional decision aid [29, 42].

According to our results, VBGs as more sophisticated and resource intensive techniques should be used to support biology of bone healing if needed and not only to replace absent vascularity. This confirms two recent studies that reported the success of NVBGs, independent of proximal pole vascularity [15, 16].

After selection of the vascularity of the bone graft, an appropriate donor site can be chosen according to the presence of deformity. If deformity is present, cortico-cancellous bone grafts from the iliac crest for NVBGs and from the medial femoral condyle for VBGs are recommended. If no deformity is present, the distal radius is the preferred donor site for both NVBGs and VBGs [36].

Previous failed surgery is a known risk factor for persistent non-union after SNU surgery. These patients were excluded in our study. For this patient group, the use of VBGs is recommended in the literature [13, 43].

### Limitations

A limitation of our study is the low patient number. Although this limits statistical power, the patient numbers are within the range of SNU literature about the topic. The effect of fracture displacement and malalignment-like flexion deformity were not investigated, as these are factors related to stability and not biology. Another limitation is the absence of clinical outcomes, which was not the main goal of the study.

## Conclusions

Nevertheless, the strong statistical significance among CT parameters, BHC, and time to union suggests that our CT classification based on bone structure and the resulting treatment algorithm may prove as a viable tool to guide bone graft selection in daily clinical practice. This study may enable further studies with the numbers necessary to demonstrate differences in clinical outcomes.
